# Ethnomedicinal Plants in Herbal Remedies Used for Treatment of Skin Diseases by Traditional Healers in Songkhla Province, Thailand

**DOI:** 10.3390/plants11070880

**Published:** 2022-03-25

**Authors:** Jongkon Saising, Katesarin Maneenoon, Oraphan Sakulkeo, Surasak Limsuwan, Friedrich Götz, Supayang Piyawan Voravuthikunchai

**Affiliations:** 1 Center of Antimicrobial Biomaterial Innovation-Southeast Asia and Natural Product Research Center of Excellence, Faculty of Science, Prince of Songkla University, Hat Yai 90110, Thailand; jongkon.sai@mfu.ac.th; 2 School of Health Science, Mae Fah Luang University, Chiang Rai 57100, Thailand; 3 Traditional Thai Medical Research and Innovation Center, Faculty of Traditional Thai Medicine, Prince of Songkla University, Hat Yai 90110, Thailand; katesarin.m@psu.ac.th (K.M.); oraphan.s@psu.ac.th (O.S.); surasak.l@psu.ac.th (S.L.); 4 Microbial Genetics, Institute of Microbiology and Infection Medicine Tübingen, University of Tübingen, 72076 Tübingen, Germany; friedrich.goetz@uni-tuebingen.de

**Keywords:** ethnomedicinal plants, herbal remedies, skin diseases, traditional healers

## Abstract

Skin disorders are a worldwide health problem that normally affect human life. A traditional healer is an important expert in researching notable medicinal plants for skin disease treatment. This study aimed to determine the traditional knowledge and the use of medicinal plants for the treatment of skin diseases among traditional healers in the Songkhla province, Thailand. The ethnobotanical information was collected from experienced traditional healers by semi-structured interviews and participant observations. Plant specimens were also collected and identified using the standard taxonomic method. The data were analyzed by interpretation and descriptive statistics. Twenty-five polyherbal formulations for the treatment of skin diseases were obtained from traditional healers with at least 10 years of experience. A total of 66 plant species in 38 families were documented. Leaves and trees were the most commonly employed plant parts and plant habits included in the herbal remedies, respectively. Fabaceae, Rubiaceae, and Zingiberaceae were the majority of the cited families. *Oryza sativa* L. and *Zingiber montanum* (J. Koenig) Link ex A.Dietr. were the most preferred plants combined in the prescriptions, which had the highest use value (UV = 0.83). The highest relative frequency of citation was represented by *Curcuma longa* L., *Eurycoma longifolia* Jack, *Knema globularia* (Lamk.) Warb, and *Senna siamea* (Lam.) Irwin & Barneby. (0.55 each). This research suggests the importance of traditional healers in the healing of skin diseases with herbal remedies. A variety of medicinal plants are used in the prescriptions for the treatment of skin disorders in the Songkhla province, in the south of Thailand. Pharmacological and toxicological activities as well as phytochemical constituents of polyherbal remedies should be further investigated to scientifically verify further applications of widely practiced herbal medicines.

## 1. Introduction

Skin disease is a global public health problem that often has physiological, psychological, and social impacts [1]. The occurrence of skin diseases usually affects human health, from newborns to elderly people. Common skin diseases impact global distribution both in resource-poor areas and advanced economic settings. Scabies and pyoderma are examples of skin diseases in resource-poor settings. Skin disorders such as atopic eczema, psoriasis, skin ulcers, and pruritus are commonly distributed in both resource-poor areas and advanced economic settings [2]. Infectious skin diseases include a variety of conditions ranging in severity. The clinical presentation and the pattern of infectious skin diseases depend on the type of causative pathogen, the layers and structures of the skin, and the underlying medical condition of the patient [3]. Although the prescribed accessible drugs have been applied for the medication of skin diseases, many adverse effects of the drugs, for example skin irritation, erythema, pruritus, staining, and skin cancers can possibly occur [4].

Natural products and traditional medicines are valuable for the treatment and prevention of various diseases [5]. They are gaining popularity as alternative treatments for common skin conditions [6]. Natural medicines from plants have been continuously prominent due to many advantages, including fewer side effects, being inexpensive, and acceptable use for a long time. Moreover, medicinal plants are also applicable raw materials for the synthesis of novel therapeutic agents. Several plants, such as *Aloe vera* (L.) Burm.f., *Azadirachta indica* A.Juss., *Calendula officinalis* L., *Cannabis sativa* L., *Portulaca amilis* Speg., and others, have been investigated for the treatment of skin diseases ranging from itching to skin cancer and have been reported to be effective in various skin diseases [7]. One hundred and six plant species are utilized in the local communities of Northern Pakistan to treat skin problems such as wound healing, skin burns, boils, pimples, inflammatory abscesses, etc. [8].

Phytochemical substances are found in plants that are utilized to treat skin problems. Plant constituents, or phytoconstituents, are divided into primary and secondary metabolites depending on their activity in a basic metabolic process. Secondary metabolites have been demonstrated to have a variety of biological effects, providing a scientific foundation for the use of herbs in traditional medicine in many ancient cultures. Secondary metabolite classes include phenolics, lipids, saponins, carbohydrates, alkaloids, and terpenes according to their chemical structures [9]. Some phytochemical compounds used to treat skin diseases, including mangiferin, lutein, curcumin, resveratrol, embelin, naringenin, quercetin, lycopene, gingerol, and apigenin, as well as their mechanism of action against skin disorders such as inflammation reduction, reduction of skin infection, wound healing, treating skin cancer, and reducing skin aging have been reviewed [10].

Traditional healers are significant for public health in Thai communities, and many individuals have confidence in the healing attributes of herbal medicine [11]. The folk healer is one of the important sources for determining the use of herbal medicine for treating people in the local area, and it will be the initiation process for searching for the prominent plants. This study focused on the Songkhla province due to its large area in the south of Thailand. Although elderly folk healers in Songkhla actively cooperated, their wisdom has been transferred in a limited manner [12]. The ethnomedicinal surveys on plants used for disease treatment in Songkhla were documented [12,13,14,15]. However, there is no systematic ethnomedicinal study attended to polyherbal prescriptions used by traditional healers for the remedy of skin disorders in this area. This study aimed to determine the traditional knowledge and examine the utilization of medicinal plants for skin disease treatment by traditional healers in the Songkhla province, in the south of Thailand.

## 2. Results and Discussion

### 2.1. Socio-Demographic Characteristics of Informants

Traditional medicines as well as herbal medicines have been used for health care in Southeast Asia and other global regions. They have been established and have developed empirical experience. This wisdom and knowledge are part of the social and cultural heritage of people and countries and can be passed from one generation to another [16]. In this study, six traditional healers participated. All professional folk healers were males. The informants were divided into three age groups, including 50–59 (16.7%), 60–69 (33.3%), and over 70 (50%) (Table 1). This finding is similar to a previous study that reported the majority of experienced healers were males aged 69–79 years old [8]. Based on education, fifty percent of the participants had graduated from primary school, while 16.7% in each group were secondary school, vocational diploma, and bachelor’s degree holders. The majority of the informants were Buddhists. In the past, Thai men had more opportunities to be educated than women. Additionally, Buddhist males had a chance to enter the monkhood. They could be the causes of men’s ability to write and read. Moreover, men take their duty of obtaining plant materials for their family’s living, leading to intensive experience in plant utilization [17].

### 2.2. Prescriptions of Polyherbal Remedies Applied for the Treatment of Skin Diseases

**Figure 1:**
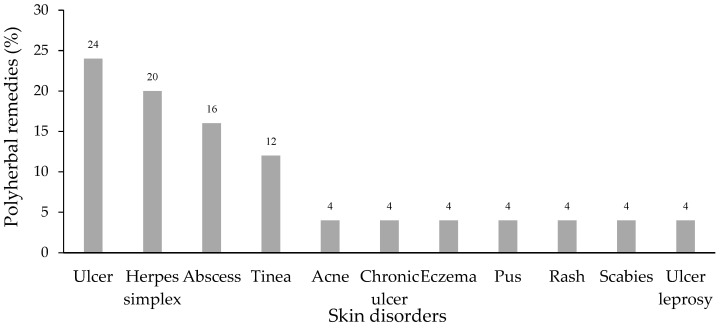
Skin disorders treated by polyherbal remedies.

Twenty-five polyherbal remedies obtained from the folk healers were used to treat skin disorders including ulcers (24%), herpes simplex (20%), abscesses (16%), and tinea (12%). The rest of the treated skin diseases are presented in Figure 1.

The information from the prescriptions in Table 2 showed that twenty-two formulations (81.8%) were applied for microbial skin infectious diseases, including bacterial infections such as ulcers, abscesses, acne, and pus, viral infections (herpes simplex), and fungal infections (tinea). The traditional healers possessed the medical expertise to formulate the herbal medicine for the treatment of each patient. The polyherbal prescription was defined by the combination of each plant at equal weight. Many diseases can be healed using one or a combination of plants as a synergistic effect. In addition, the plant’s scientific name, local name, family, and plant part used are also shown in Table 2.

**Figure 2:**
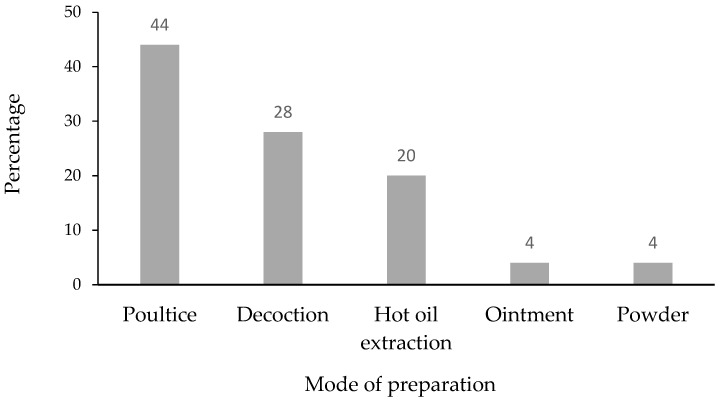
Mode of preparation of polyherbal prescriptions used by traditional healers.

The traditional preparations of the remedies were made by poultice (44%), decoction (28%), hot oil extraction (20%), ointment (4%) and powder (4%) as shown in Figure 2. A variety of medicinal plant preparation methods for skin disorder treatment, such as powder, paste, oil, infusion, decoction, and concoction, have been documented in many ethnobotanical surveys [8,18,19]. The poultice was the most famous preparation method applied to the patients in this study. It might be due to the convenient preparation used for topical skin disease treatment.

### 2.3. The Habits of Medicinal Plants and the Plant Part Used

**Figure 3:**
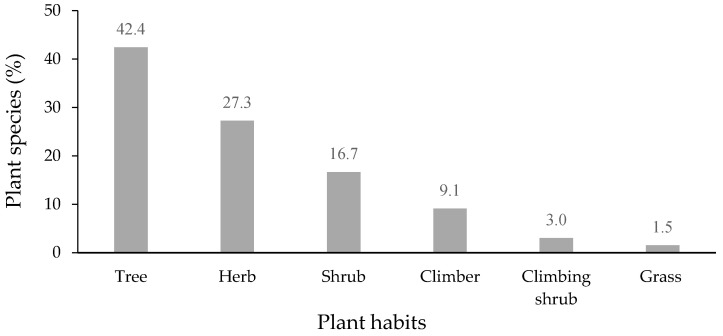
Habit of plants in polyherbal remedies used for the treatment of skin diseases (*n* = 66).

All plant species in the 25 polyherbal remedies were classified into six habits (Figure 3). Trees (42.4%) were found to be the most commonly used plant habit included in herbal remedies, followed by herbs (27.3%), shrubs (16.7%), and climbers (9.1%). Trees were the most preferable plant habit used in polyherbal prescriptions, which was consistent with the habits of medicinal plants from a previous study in the Songkhla province [12].

**Figure 4:**
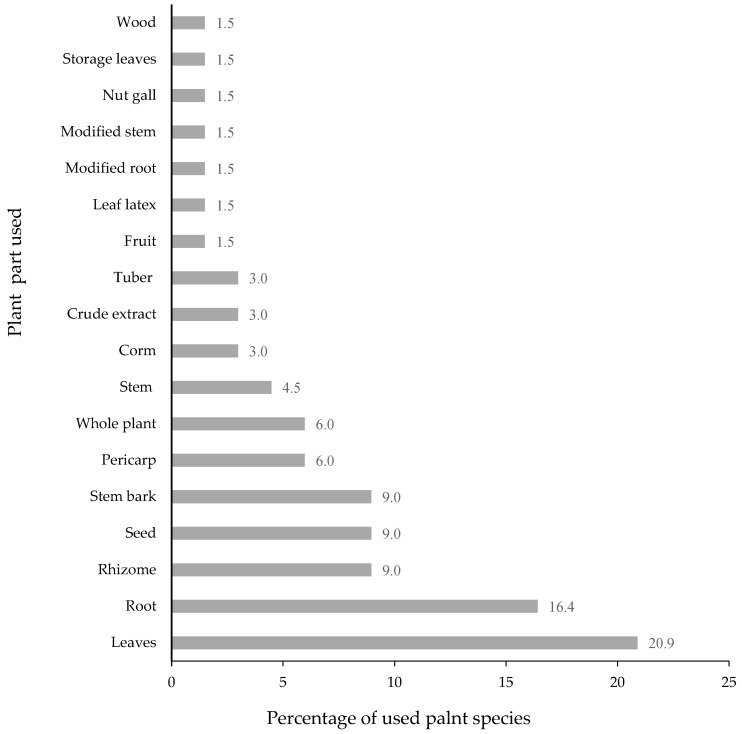
Plant part used in herbal remedies for skin diseases (*n* = 66).

Sixteen different plant parts were used in the polyherbal formulations for skin infection. Leaves were the most frequently used part (20.9%), followed by roots (16.4%), rhizomes (9.0%), seeds (9.0%), and stem bark (9%). The rest of the parts used of the plants are shown in Figure 4. In many reports [8,20], as well as in this study, leaves were the major plant part used for skin disease treatment. It could be due to the fact that the collection of leaves is easier than that of other parts, such as roots, seeds, bark, and rhizomes, and they are harvested every season. Another reason might be that leaves are soft and the chemical contents might be readily extracted [12].

### 2.4. A Variety of Plant Materials Used in Polyherbal Remedies for Skin Disorders

Totally, 66 plant species and one animal material (*Sepia* spp. cuttlebone) were included in the 25 prescriptions of herbal remedies. The plants were classified into 61 genera and 38 families (Table 2). In regard to the numbers of plant species used in the families, Fabaceae, Rubiaceae, and Zingiberaceae (7.6%) were the most notable families, followed by Acanthaceae (6.1%), as shown in Table 3. The plants in the Fabaceae family included *Acacia catechu* (L.f.) Willd., *Entada rheedii* Spreng., *Pterocarpus indicus* Willd., *Senna alata* (L.) Roxb., and *Senna siamea* (Lam.) Irwin & Barneby. *Mitragyna speciosa* (Roxb.) Korth., *Uncaria gambir* (Hunter) Roxb., *Prismatomeris tetrandra* (Roxb.) K. Schum, *Hydnophytum formicarium* Jack, and *Ceriscoides turgida* (Roxb.) Tirveng. were in Rubiaceae, while those in the Zingiberaceae consisted of *Zingiber montanum* (J.Koenig) Link ex A.Dietr., *Curcuma zedoaria* (L.) Roscoeex Sm., *Curcuma longa* L., *Zingiber zerumbet* (L.) Roscoeex Sm., and *Curcuma aromatica* Salisb. Fabaceae and Zingiberaceae are well known among plants used in traditional medicine. The study of medicinal plants used in Thai traditional medicine in modern healthcare services reported 89 medicinal plant species. The plants belonged to 37 families, with the highest numbers of medicinal plant species being Zingiberaceae (11 species) and Fabaceae (10 species) [21]. Similarly, Zingiberaceae, Fabaceae, and Rubiaceae were the major families of plant species used and cited by traditional healers in the Patthalung province, in the south of Thailand [11]. In addition, five plant species in the Fabaceae family and three species in the Rubiaceae family were used in herbal remedies for skin diseases treated by a folk healer in the Songkhla province [22].

### 2.5. Preferred Plants Used in Polyherbal Recipes for Skin Disorder Treatment

The preferred medicinal plants used in polyherbal remedies for skin diseases and their pharmacological activities are presented in Table 4. According to the quantitative analysis, *Oryza sativa* L. and *Zingiber montanum* (Koenig) Link ex Dietr. show the highest use value with 0.83, followed by *Nicotiana tabacum* L. with a use value of 0.67. *Oryza sativa* L. was applied for herpes simplex and abscess treatment, while *Zingiber montanum* (Koenig) Link ex Dietr. was used for treating tinea, acne, and ulcer leprosy, which is similar to *Nicotiana tabacum* L. Other important plants were *Curcuma longa* L., *Eurycoma longifolia* Jack, *Knema globularia* (Lamk.) Warb, and *Senna siamea* (Lam.) Irwin & Barneby. with a use value of 0.50. The species with a use value of 0.33 are listed in Table 4. Other plants (50 species) exhibited the UV of 0.17. In previous reports, the seed of *Oryza sativa* L. was frequently used in skin treatment. *Oryza sativa* L. contained high levels of anthocyanin polyphenols, which presented beneficial effects on health owing to their antioxidant properties. Anthocyanin from *Oryza sativa* L. exhibited anti-inflammatory properties and anti-aging activity by modulating type I collagen gene expression and suppressing H_2_O_2_-induced NF-κB activation in skin fibroblasts [23]. The crude extract, alkaloids, flavonoids, and saponins from *Oryza sativa* L. showed antibacterial effects against multidrug resistant *Staphylococcus aureus* [24]. In addition, the antimicrobial activity of *Oryza sativa* L. against fungi [25] and viruses [26] has been revealed. Cream containing niosomes loaded with purple glutinous rice (*Oryza sativa* L.) extract possessed anti-aging activity on human skin [27]. The antioxidative and immunomodulatory properties of *Oryza sativa* L. crude extract reduced the severity of psoriasis [28]. *Zingiber montanum* (Koenig) Link ex Dietr., another one of the most frequently used plants in this study, has been previously investigated for its phytochemicals and pharmacological activity. Numerous bioactive phytochemicals were discovered in the rhizomes of *Zingiber montanum* (Koenig) Link ex Dietr. including alkaloids, saponins, tannins, flavonoids, terpenoids, phenolic compounds, phlobatannins, steroids, and glycosides [29,30]. The essential oil of *Zingiber montanum* (Koenig) Link ex Dietr. rhizome exhibited antifungal activity against *Candida albicans* [31]. (E)-8(17),12-labdadiene-15,16-dial, zerumbol, zerumbone, buddledone A, furanodienone, germacrone, borneol, and camphor were isolated from the rhizomes of *Zingiber montanum*. Among these terpenes, (E)-8(17),12-labdadiene-15,16-dial and zerumbol exhibited antibacterial activity against a number of clinical isolates of multi-drug-resistant (MDR) and methicillin- resistant *Staphylococcus aureus* (MRSA) [32]. Zerumbone, a sesquiterpenoid, is one of the major compounds in the essential oils and rhizomes of *Zingiber montanum*. Furthermore, zerumbone-treated wound sections showed greater tissue regeneration and more fibroblasts, possibly through the enhanced expression of VEGF, TGF-β1 and collagen IV [33]. Cysteine protease glycoprotein, purified from *Zingiber montanum* rhizome, showed antioxidant activity in biochemical systems and THP-1 cells [34], and anti-inflammatory activity [35]. The leaf of *Nicotiana tabacum* L. was the preferred component in the formulations, and it possessed many biological activities. The different extracts of *Nicotiana tabacum* L. leaves contain the phytochemical constituents of alkaloids, phenolic compounds, tannins, flavonoids, steroids, terpenoids, cardiac glycosides, essential oils, resins, saponins, quinones, and polypeptides [36]. Antimicrobial activity was observed in the ethyl acetate extract of *Nicotiana tabacum* L. against *Staphylococcus aureus*, *Pseudomonas aeruginosa*, *Klebsiella pneumoniae*, and biofilm-forming *Escherichia coli* and *Klebsiella* species. The most common phytochemical components found in the ethyl acetate extract were 3, 4, 5,6-tetrahydro-1, 3-dimethyl-2(1h)-pyrimidinone, pyridine, 3-(1-methyl-2-pyrrolidinyl)-, (S)-, isododecane, *n*-pentadecane, and tetradecylaldehyde. The antibacterial property demonstrated could be due to pyridine, 3-(1-methyl-2-pyrrolidinyl)-(S), the major compound detected, with a broad spectrum of activity [37]. Six sesquiterpenes, including tabasesquiterpenes A−C, balsamiferine B, samboginone, and ent-4(15)-eudesmen-1α,11-diol were isolated from the leaves of *Nicotiana tabacum* L. Tabasesquiterpenes B exhibited high antiviral activity with an inhibition rate of 35.2%. The other compounds also demonstrated antiviral activity with inhibition rates ranging from 20.5–28.6% [38]. *Nicotiana tabacum* L. leaf cow urine extract was found to have potential anti-dandruff activity against a causative agent, *Malassezia furfur* [39].

The plants that were commonly used by traditional healers for the treatment of skin diseases were illustrated by relative frequency citation (RFC) (Table 4). The RFC values ranged from 0.17–0.50. The highest RFC value (0.50) was reported for *Curcuma longa* L., *Eurycoma longifolia* Jack, *Knema globularia* (Lamk.) Warb, and *Senna siamea* (Lam.) Irwin & Barneby. Other high RFC species included *Curcuma zedoaria* (L.) Roscoeex Sm., *Datura metel* L., *Garcinia mangostana* L., *Nicotiana tabacum* L., *Oryza sativa* L., *Punica granatum* L., *Quercus infectoria* Oliv., and *Tiliacora triandra* (Colebr.) Diels. The RFC value of 0.17 belonged to the rest of the plants (53 species). Curcumin and derivatives from *Curcuma longa* L. exhibited biological activities. Curcumin showed anti-bacteria, anti-HIV, antioxidant, anti-inflammatory, and anti-tumor activity. Demethoxy curcumin and bisdemethoxy curcumin had antioxidant activity, while sodium curcuminate showed anti-inflammation [40]. The presence of high levels of curcuminoids and other compounds in MeOH extracts from *Curcuma longa* L. reflected the potency of antioxidant activity [41]. Curcumin and its derivatives, gallium-curcumin and Cu-curcumin, exhibited remarkable antiviral effects on herpes simplex virus type 1 (HSV-1) in cell culture [42]. Ethanolic extract of *Curcuma longa* L. rhizomes was found to have better and faster wound healing activity than the standard drug povidone iodine ointment on the excision wound model [43]. The hydroalcoholic extract of *Eurycoma longifolia* Jack showed significant antioxidant and anti-inflammatory activity [44]. Phenolic compounds, flavonoids, terpenoids, alkaloids, protein, and cardiac glycosides were presented in the extracts from the stem and root of *Eurycoma longifolia* Jack. The extracts showed antimicrobial activity against *Bacillus cereus*, *Staphylococcus aureus*, and *Aspergillus niger* [45]. Two quassinoid compounds including 14,15 β-dihydroxyklaineanone and eurycomanone had strong antiproliferative activities against all tested cancer cell lines including KATO III (stomach cancer), HCT-15 (colon cancer), Colo205 (colon cancer), HepG2 (hepatoma), PC-3 (prostate cancer), HL-60 (promyelocytic leukemia), and Jurkat (acute T cell leukemia) [46]. Knecorticosanone B and malabaricone D from the fruits of *Knema globularia* (Lamk.) Warb, exhibited a moderate cytotoxic effect against Hep-G2, MCF-7 and SK-LU-1 cell lines [47]. Giffithane, a compound isolated and characterized from the roots of *Knema globularia* (Lamk.) Warb, showed strong cytotoxicity against the NCI-H187 and MCF-7 cell lines with IC_50_ values of 3.08 and 6.68 mg/mL, respectively [48]. Six compounds, knecorticosanones C–H were isolated from the fruits of Knema globularia (Lamk.) Warb. Knecorticosanones C exhibited the most cytotoxicity against HepG2 and KKU-M156 cell lines [49]. Chloroform and 95% ethanolic extracts of *Senna siamea* (Lam.) Irwin & Barneby leaves had good antifungal activity against *Candida albicans* and *Aspergillus niger*. Petroleum ether extracts of leaves were found to be very active against *S. aureus* [50]. Senna siamea (Lam.) Irwin & Barneby extract contained alkaloid, anthraquinone, saponin, tannin, phenol, steroid, flavonoid, terpenoid, and glycosides, according to phytochemical screening. *Senna siamea* (Lam.) Irwin & Barneby leaf extracts are effective against *Klebsiella pneumoneae*, *Salmonella typhi*, *Shigella* spp., *Escherichia coli*, and *Pseudomonas aeruginosa*. The antibacterial activities of the extracts were expected due to the presence of bioactive compounds, which were dissolved in the solvents [51]. An aqueous extract of *Senna siamea* (Lam.) Irwin & Barneby leaves showed interesting activity against inflammation. Alkaloids, polyphenols, terpenoids, steroids, anthraquinones, cardiotonic glycosides, and anthocyanins have been identified in this plant [52]. All the other preferable plants combined in the herbal formulations exhibited antimicrobial, anti-oxidant, and anti-inflammatory activities as shown in Table 4. Interestingly, wound healing activity was reported in notable plants including *Curcuma longa* L [43], *Curcuma zedoaria* (L.) Roscoeex Sm. [53], *Aloe vera* (L.) Burm.f. [54], *Garcinia mangostana* L. [55], *Punica granatum* L. [56], gall of *Quercus infectoria* Oliv. [57], and *Tinospora crispa* (L.) Miers ex Hook.f. [58]. A variety of plants indicated in this study exhibited dermatological healing properties.

## 3. Methods

### 3.1. Study Area

The Songkhla province is located in the eastern part of the south of Thailand between latitude 6°17′–7°56′ N and longitude 100°01′–101°06′ E. The province is on the Malay Peninsula, on the coast of the Gulf of Thailand. The height above mean sea level is 4 m. It is approximately 950 km from Bangkok, the capital city. It covers 7393.889 km^2^. The north is connected to Nakhon Si Thammarat and Phatthalung provinces, while the east borders on the Gulf of Thailand. The neighboring provinces in the south are Yala and Pattani in Thailand and Kadah and Perlis in Malaysia. Phatthalung and Satun provinces are the neighbors in the west. It had a total population of 1,432,628 in 2018 with 63.71% being Buddhists, 33.16% being Muslims, and 3.19% being Christians and Hindus. The weather conditions in Songkhla are influenced by the southwest and northeast monsoons. The average annual temperature is 27.76 °C. The annual rainfall is about 3,434.9 mm, with a relative average humidity of 79.93% [114]. The study was conducted in five districts in Songkhla province, including Muang Songkhla, Chana, Rattaphum, Khuan Niang, and Singhanakhon.

### 3.2. Informants

In the present study, all traditional healers were selected from their extensive experience and actively practiced patient treatment. All the informants had experience of at least 10 years. According to the intensive criteria, six traditional healers were chosen. All the professional folk healers were males, 54–74 years old. Based on education, three of the healers had graduated from primary school, while one in each group was a secondary school, vocational diploma, and bachelor’s degree holder. Five of the informants were Buddhist, and one of them was Muslim (Table 1).

### 3.3. Ethnobotanical Data Collection

Ethnobotanical information about skin diseases was obtained from local herbal healers. Before interviewing, the purposes of the study were distinctly explained to the traditional healers and their family members, and verbal informed consent was obtained.

The informants were interviewed using questionnaires and conversations. The interview was performed to investigate the prescriptions for skin disease treatment. Data on the plant’s local names, plant parts used, skin diseases treated with herbal remedies, mode of use, and administration were gathered. Field trips to the sites where the traditional herbal healers normally go to harvest the plants were carried out. Plant materials were collected and processed according to the standard taxonomic method [115]. The scientific names of the plants in the polyherbal formulations were identified according to the principle of plant taxonomy using the Flora of Thailand and the related literature from neighboring areas. The accepted names were verified against The Plant List (2013) [116]. Voucher specimens were deposited at the Faculty of Thai Traditional Medicine, Prince of Songkla University, Songkhla, Thailand. However, some medicinal plants were not obtained from nature because they were exotic or not distributed in the study location. Therefore, they were purchased from the drug stores following the healers’ suggestion, and for their scientific names, we referred to authentic books in traditional Thai pharmacy.

### 3.4. Data Analysis

The plants were analyzed regarding their habits of plants, and plant parts used in polyherbal remedies for treatment of skin diseases, frequency in families, and quantitative ethnobotany analysis including use value (UV) and relative frequency of citation (RFC).

### 3.5. Use Value (UV)

Use value was calculated based on the number of uses and the number of people citing a given plant. It indicated the most significant plant species, recognized by a given population [117].

UV = u/N (1)

where u is the total number of use reports stated by participants for a given species, and N is the whole number of participants. UV is normally “1” if there are more usages, and “0” if there are fewer usages reported for plant species.

### 3.6. Relative Frequency of Citation (RFC)

RFC was analyzed to intricate the knowledge of traditional flora about usage of therapeutic flora in the study site.

RFC = FC/N (0 < RFC < 1)(2)

where RFC is denoted by relative frequency citation, FC (frequency of citation) is the number of participants mentioning the plant species and N is total number of participants [118].

## 4. Conclusions

Currently, the Thai government has a policy to promote and develop the use of traditional medicines. Thai traditional medicines as well as drugs developed from medicinal plants are included in the national list of essential medicines. The traditional healer is one of the most essential sources for determining the usage of herbal medicine for treating individuals in the community. The results from this study represent the polyherbal remedies used by experienced folk healers for the treatment of skin disorders in the Songkhla province, in the south of Thailand. Ethnopharmacological expertise is abundant among traditional healers. They have significant knowledge of many plant species used for a variety of skin disorder treatment. The utilization of medicinal plants was widespread in the prescriptions, with 66 species in 38 families. The poultice was the most frequent method of administration. The most prominent plant families were Fabaceae, Rubiaceae, and Zingiberaceae. The highest use values were reported for *Oryza sativa* L. and *Zingiber montanum* (Koenig) Link ex Dietr. Based on RFC, the highest was found for *Curcuma longa* L., *Eurycoma longifolia* Jack, *Knema globularia* (Lamk.) Warb, and *Senna siamea* (Lam.) Irwin & Barneby. Although local treatment of herbal prescriptions and ethnobotanical surveys are underway, more research on phytochemicals and their pharmacological activities is needed to ensure the application of polyherbal prescriptions used by traditional healers, as well as product development in herbal medicine for the treatment of skin diseases, in order to promote the sustainable and safe use of natural resources.

## Figures and Tables

**Table 1:** Demographical characteristics of experienced traditional healers in the Songkhla province, Thailand.

Demographical Characteristics	Number of Informants (*n* = 6)
Sex	
Male	6
Age (years)	
50–59	1
60–69	2
>70	3
Education level	
Primary school	3
Secondary school	1
Vocational diploma	1
Bachelor’s degree	1
Religion	
Buddhism	5
Islam	1

All informants had at least 10 years of experienced.

**Table 2:** Polyherbal remedies used by traditional healers for the treatment of skin diseases in the Songkhla province, Thailand.

Prescription	Skin Disorder	Scientific Name/Voucher Number	Local Name	Family	Parts Used	Formulation Form
1. SKHS1-SC1	Herpes simplex	*Jasminum sambac* (L.) Aiton/ N. Kiankhun 009	Ma li	Oleaceae	Root	Poultice
		*Oryza sativa* L./ N. Kiankhun 010	Khao	Poaceae	Seed	
2. SKHS2-SC2	Herpes simplex	*Mitragyna speciosa* (Roxb.) Korth./ N. Kiankhun014	Kra tom	Rubiaceae	Leaf	Poultice
3. SKHS3-SC3	Herpes simplex	*Glycosmis pentaphylla* (Retz.) DC./ N. Kiankhun 015	Khoei tai	Rutaceae	Root	Poultice
		*Oryza sativa* L./ N. Kiankhun 010	Khao	Poaceae	Seed	
4. SKHS4-SC4	Herpes simplex	*Mirabilis jalapa* L./ N. Kiankhun 016	Ban yen	Nyctaginaceae	Root	Poultice
		*Oryza sativa* L./ N. Kiankhun 010	Khao	Poaceae	Seed	
5. SKHS5-SC5	Herpes simplex	*Euphorbia hirta* L./ N. Kiankhun 017	Nam num rat cha si	Euphorbiaceae	Whole plant	Poultice
		*Oryza sativa* L./ N. Kiankhun 010	Khao	Poaceae	Seed	
6. SKEC1-SC6	Eczema	*Tinospora crispa* (L.) Miers ex Hook.f. & Thomson/N. Kiankhun 018	Bor ra pet	Menispermaceae	Stem	Poultice
		*Psidium guajava* L./ N. Kiankhun 019	Pha rang	Myrtaceae	Leaf	
		*Areca catechu* (L.f.) Willd./ N. Kiankhun 020	Mak	Arecaceae	Seed	
		*Piper betle* L./ N. Kiankhun 021	Plu	Piperaceae	Leaf	
7. SKTI1-SC7	Tinea	*Quercus infectoria Oliv.* *	Ben ga nee	Fagaceae	Nut gall *	Hot oil extraction
		*Acacia catechu (L.f.) Willd.* *	Si siad tai	Fabaceae	Crude extract	
		*Uncaria gambir* (Hunter) Roxb. *	Si siad tet	Rubiaceae	Crude extract	
		*Zingiber montanum* (Koenig) Link ex Dietr./N. Kiankhun 025	Phlai	Zingiberaceae	Rhizome	
		*Aloe vera* (L.) Burm.f. *	Wan hang cho ra khe	Xanthorrhoeaceae	Leaf’s latex (resin)	
		*Anacardium occidentale* L./ N. Kiankhun 044	Ma muang him ma pan	Anacardiaceae	Pericarp	
		*Entada rheedii* Spreng./ N. Kiankhun 028	Sa ba	Fabaceae	Seed	
		*Knema globularia* (Lamk.) Warb/ N. Kiankhun 029	Han	Myristicaceae	Seed	
		*Barringtonia acutangula* (L.) Garetn./ N. Kiankhun 030	Chik na	Lecythidaceae	Stem bark	
8. SKTI2-BK8	Tinea	*Datura metel* L./ N. Kiankhun 032	Lum pong ka sa lak	Solanaceae	Seed	Ointment
		*Pterocarpus indicus* Willd./ N. Kiankhun 033	Pra du	Fabaceae	Stem bark	
		*Hopea odorata* Roxb./ N. Kiankhun 034	Ta kian	Dipterocarpaceae	Stem bark	
		*Knema globularia* (Lamk.) Warb/ N. Kiankhun 029	Han	Myristicaceae	Seed	
		*Tiliacora triandra* (Colebr.) Diels./ N. Kiankhun 036	Ya nang	Menispermaceae	Stem	
		*Cratoxylun cochinchinense* (Lour.) Blume/N. Kiankhun 037	Tio	Hypericaceae	Stem bark	
		*Curcuma zedoaria* (L.) Roscoe ex Sm./ N. Kiankhun 094	Ka min ooi	Zingiberaceae	Rhizome	
9. SKTI3-YH9	Tinea	*Typhonium trilobatum* Schott./ N. Kiankhun 039	Ut ta pid	Araceae	Corm	Hot oil extraction
		*Alocasia longiloba* Miq./ N. Kiankhun040	O nok	Araceae	Corm	
		*Rhinacanthus nasutus* (L.) Kurz./ N. Kiankhun 041	Thong pan chang	Acanthaceae	Leaf	
		*Nicotiana tabacum* L./ N. Kiankhun042	Ya sueb	Solanaceae	Leaf	
10. SKLY1-SC10	Ulcer	*Anacardium occidentale* L./ N. Kiankhun044	Ma muang him ma pan	Anacardiaceae	Pericarp	Hot oil extraction
		*Zingiber montanum* (Koenig) Link ex Dietr./N. Kiankhun025	Phlai	Zingiberaceae	Rhizome	
		*Aloe vera* (L.) Burm.f. *	Wan hang cho ra khe	Xanthorrhoeaceae	Leaf’s latex (resin)	
11. SKLY2-BK11	Ulcer	*Prismatomeris tetrandra* (Roxb.) K. Schum/N. Kiankhun 048	Kra duk kai	Rubiaceae	Root	Decoction
		*Eurycoma longifolia* Jack/ N. Kiankhun 049	Lai phueak	Simaroubaceae	Root	
		*Dianella ensifolia* (L.) DC./ N. Kiankhun 050	Ya nu ton	Xanthorrhoeaceae	Root	
		*Arcangelisia flava* (L.) Merr./ N. Kiankhun 051	Ka min kruea	Menispermaceae	Stem	
12. SKPU1-SC12	Pus	*Lasia spinosa* (L.) Thw./ N. Kiankhun 053	Pak nam	Araceae	Rhizome	Decoction
		*Nicotiana tabacum* L./ N. Kiankhun 042	Ya sueb	Solanaceae	Leaf	
13. SKAB1-BK13	Abscess	*Ceiba pentandra* (L.) Gaertn./ N. Kiankhun056	Nun	Malvaceae	Leaf	Poultice
		*Curcuma longa* L./ N. Kiankhun 057	Ka min chan	Zingiberaceae	Rhizome	
		*Senna siamea* (Lam.) Irwin & Barneby./N. Kiankhun 058	Khi lek	Fabaceae	Leaf	
		*Oryza sativa* L./ N. Kiankhun 010	Khao	Poaceae	Seed	
14. SKAB2-SC14	Abscess	*Garcinia mangostana* L./ N. Kiankhun 061	Mung kud	Clusiaceae	Pericarp	Poultice
		*Nicotiana tabacum* L./ N. Kiankhun 042	Ya sueb	Solanaceae	Leaf	
15. SKAB3-SC15	Abscess	*Tinospora crispa* (L.) Miers ex Hook.f. & Thomson/N. Kiankhun 018	Bor ra pet	Menispermaceae	Stem	Decoction
		*Eurycoma longifolia* Jack/ N. Kiankhun 049	Lai phueak	Simaroubaceae	Root	
		*Olax psittacorum* (Willd.) Vahl/ N. Kiankhun 065	Nam chai krai	Oleaceae	Stem bark	
16. SKAB4-BP16	Abscess	*Smilax corbularia* Kunth. *	Khoa yen neua	Smilacaceae	Tuber	Decoction
		*Smilax glabra* Wall.ex Roxb.	Khoa yen tai	Smilacaceae	Tuber	
		*Senna siamea* (Lam.) Irwin & Barneby./N. Kiankhun 058	Khi lek	Fabaceae	Wood	
		*Hydnophytum formicarium* Jack/N. Kiankhun 070	Hua roi ru	Rubiaceae	Modified stem	
		*Eurycoma longifolia* Jack/ N. Kiankhun 049	Lai phueak	Simaroubaceae	Root	
		*Clerodendrum inerme* (L.) Gaertn./ N. Kiankhun 072	Sam ma nga	Lamiaceae	Root	
		*Acanthus ebracteatus* Vahl./ N. Kiankhun 073	Ngueak pla mor	Acanthaceae	Whole plant	
17. SKTD1-YH17	Ulcer	*Sepia* spp./*Sepiella* spp. (animal) *	Kra dong muk	Sepiidae	Cuttlebone	Powders
		*Quercus infectoria* Oliv.*	Ben ga nee	Fagaceae	Nut gall	
		*Punica granatum* L./ N. Kiankhun 077	Tub tim	Punicaceae	Pericarp	
18. SKCD1-SC1	Rash	*Citrus maxima* (Burm.) Merrill./ N. Kiankhun 079	Som O	Rutaceae	Pericarp	Decoction
		*Senna siamea* (Lam.) Irwin & Barneby./N. Kiankhun 058	Khi lek	Fabaceae	Leaf	
		*Azadirachta indica* A. Juss./ N. Kiankhun 081	Sa dao	Meliaceae	Leaf	
		*Cardiospermum halicacabum* L./ N. Kiankhun 082	Kok kra oom	Sapindaceae	Whole plant	
		*Senna alata* (L.) Roxb./ N. Kiankhun 083	Chum hed tet	Fabaceae	Leaf	
19. SKSC1-SC19	Chlonic ulcer	*Annona squamosa* L. (L.) Miers ex Hook.f.& Thomson/N. Kiankhun 083	Noi na	Annonaceae	Root	Decoction
		*Phyllanthus emblica* L./ N. Kiankhun 085	Ma kam pom	Phyllanthaceae	Root	
		*Streblus asper* Lour./ N. Kiankhun 086	Khoi	Moraceae	Root	
		*Sandoricum koetjape* (Burm. f.) Merr./N. Kiankhun 086	Kra ton	Meliaceae	Root	
		*Barringtonia acutangula* (L.) Garetn./N. Kiankhun 030	Chik na	Lecythidaceae	Stem bark	
20. SKCD2-SC20	Ulcer	*Clinacanthus nutans* (Burm.f.) Lindau/N. Kiankhun 089	Pha ya yor	Acanthaceae	Leaf	Poultice
		*Carallia brachiata* (Lour.) Merr./ N. Kiankhun 090	Chiang phra nang ae	Rhizophoraceae	Leaf	
21. SKTD2-BP21	Ulcer	*Garcinia mangostana* L./ N. Kiankhun061	Mung kud	Clusiaceae	Pericarp	Poultice
		*Punica granatum* L./N. Kiankhun077	Tub tim	Punicaceae	Pericarp	
		*Curcuma zedoaria* (L.) Roscoeex Sm./ N. Kiankhun094	Ka min ooi	Zingiberaceae	Rhizome	
		*Curcuma longa* L./N. Kiankhun 057	Ka min chan	Zingiberaceae	Rhizome	
		*Zingiber montanum* (Koenig) Link ex Dietr./N. Kiankhun 025	Phlai	Zingiberaceae	Rhizome	
		*Syzygium* cf. *claviflorum* (Roxb.) A.M. Cowan& Cowan/N. Kiankhun097	Wa	Myrtaceae	Stem bark	
22. SKTD3-KK22	Ulcer	*Combretum* cf. *quadrangulare* Kurz/ N. Kiankhun 099	Sang kae	Combretaceae	Leaf	
		*Quisqualis indica* L./ N. Kiankhun100	Leb muea nang	Combretaceae	Leaf	
		*Phyllanthus amarus* L./ N. Kiankhun 101	Luk tai bai	Phyllanthaceae	Whole plant	
23. SKSB1-PB23	Scabies	*Knema globularia* (Lamk.) Warb/ N. Kiankhun 029	Han	Myristicaceae	Seed	Hot oil extraction
		*Tiliacora triandra* (Colebr.) Diels./ N. Kiankhun 036	Ya nang	Menispermaceae	Stem	
		*Ceriscoides turgida* (Roxb.) Tirveng. *	Kra bian	Rubiaceae	Fruit	
		*Hydnocarpus anthelminthicus* Pierre ex Laness./N. Kiankhun 106	Kra bao	Flacourtiaceae	Seed	
		*Allium sativum* L./ N. Kiankhun 107	Kra tiam	Alliaceae	Storage leaf	
24. SKAN1-SC25	Acne	*Justicia adhatoda* L./ N. Kiankhun 109	Sa niad	Acanthaceae	Leaf	Poultice
		*Zingiber montanum* (Koenig) Link ex Dietr./N. Kiankhun 025	Phlai	Zingiberaceae	Rhizome	
		*Zingiber zerumbet* (L.) Smith./ N. Kiankhun 111	Ka thue	Zingiberaceae	Rhizome	
		*Curcuma longa* L./ N. Kiankhun057	Ka min chan	Zingiberaceae	Rhizome	
		*Curcuma aromatica* Salisb./ N. Kiankhun 113	Wan nang kum	Zingiberaceae	Rhizome	
25. SKLP1-SC25	Ulcer leprosy	*Nicotiana tabacum* L./ N. Kiankhun 042	Ya sueb	Solanaceae	Leaf	Hot oil extraction
		*Zingiber montanum* (Koenig) Link ex Dietr./N. Kiankhun 025	Phlai	Zingiberaceae	Rhizome	
		*Curcuma zedoaria* (L.) Roscoeex Sm./N. Kiankhun 094	Ka min ooi	Zingiberaceae	Rhizome	
		*Stemona tuberosa* Lour./ N. Kiankhun 117	Non tai yak	Stemonaceae	Modified root	
		*Datura metel* L./ N. Kiankhun 032	Lum pong	Solanaceae	Seed	

* Materia medica was bought from the drug store and the scientific name was referred to valid books in traditional Thai pharmacy.

**Table 3:** Percentage of plant species in 38 families in polyherbal remedies used for skin diseases.

Families	Percent of Species	Families	Percent of Species
Fabaceae	7.6	Clusiaceae	1.5
Rubiaceae	7.6	Dipterocarpaceae	1.5
Zingiberaceae	7.6	Euphorbiaceae	1.5
Acanthaceae	6.1	Fagaceae	1.5
Araceae	4.5	Flacourtiaceae	1.5
Menispermaceae	4.5	Hypericaceae	1.5
Combretaceae	3.0	Lamiaceae	1.5
Meliaceae	3.0	Lecythidaceae	1.5
Myrtaceae	3.0	Malvaceae	1.5
Oleaceae	3.0	Moraceae	1.5
Phyllanthaceae	3.0	Myristicaceae	1.5
Rutaceae	3.0	Nyctaginaceae	1.5
Smilacaceae	3.0	Piperaceae	1.5
Solanaceae	3.0	Poaceae	1.5
Xanthorrhoeaceae	3.0	Punicaceae	1.5
Alliaceae	1.5	Rhizophoraceae	1.5
Anacardiaceae	1.5	Sapindaceae	1.5
Annonaceae	1.5	Simaroubaceae	1.5
Arecaceae	1.5	Stemonaceae	1.5

**Table 4:** Preferred plants used in polyherbal remedies for skin diseases and their pharmacological activities.

Scientific Name	UV	RFC	Part Used	Pharmacological Activity
*Oryza sativa* L.	0.83	0.33	Seed	Anti-oxidant and anti-inflammatory activity [23] Antibacterial activity [24] Antifungal activity [25] Antiviral effect [26] Anti-aging activity [27] Reduction of psoriasis severity [28] Anti-arthritic Activity [59]
*Zingiber montanum* (Koenig) Link ex Dietr.	0.83	0.33	Rhizome	Antioxidant activity [34] Anti-inflammatory activity [35] Antibacterial activity [32] Antifungal activity [31] Anti-ulcer property [60] Anticancer activity [61]
*Nicotiana tabacum* L.	0.67	0.33	Leaf	Antibacterial and antifungal activity [62] Antiviral activity [38] Anti-oxidant activity [63] Anti-dandruff [39] Anti-aphthous activity [64]
*Curcuma longa* L.	0.50	0.50	Rhizome	Antibacterial activity [65] Antifungal activity [66] Antiviral activity [67] Antioxidant activity [41] Anti-inflammatory activity [68] Wound healing activity [43] Hyaluronidase inhibitor activity [69]
*Eurycoma longifolia* Jack	0.50	0.50	Root	Antioxidant and anti-inflammatory activity [44] Antibacterial and antifungal activity [45,70] Anticancer activity [46] Tyrosinase inhibition activity [71]
*Knema globularia* (Lamk.) Warb	0.50	0.50	Seed	Cytotoxicity activity [47] (fruit), [48] (root)
*Senna siamea* (Lam.) Irwin & Barneby.	0.50	0.50	Leaf	Antibacterial and antifungal activity [50,51] Anti-inflammatory and analgesic activity [52] Antioxidant activity [72]
*Curcuma zedoaria* (L.) Roscoeex Sm.	0.33	0.33	Rhizome	Antibacterial and anti-inflammatory activity [73] Antifungal activity [74] Anti-oxidant activity [75] Wound healing activity [53] Antitumor activity [76]
*Datura metel* L.	0.33	0.33	Seed	Antibacterial activity [77] Antifungal activity [78] Antiviral activity [79] Antioxidant activity [80]
*Garcinia mangostana* L.	0.33	0.33	Pericarp	Antibacterial activity [81] Antifungal activity [82] Antiviral activity [83] Antioxidant activity [84] Anti-inflammatory activity [81] Anti-skin cancer property [85] Remedial effect on skin conditions [86] Cell proliferation and Wound healing activity [55] Increased skin collagen thickness and density [87]
*Punica granatum* L.	0.33	0.33	Pericarp	Antibacterial activity [88] Antifungal activity [89] Antiviral activity [90] Antioxidant activity [91] Anti-inflammatory activity [92] Anti-melanoma activity [93] Wound healing activity [56]
*Quercus infectoria* Oliv.	0.33	0.33	Gall	Antibacterial activity [94] Antifungal activity [95] Antioxidant activity [96] Anti-inflammatory activity [97] Wound healing activity [57,98]
*Tiliacora triandra* (Colebr.) Diels.	0.33	0.33	Stem	Antibacterial and antifungal activity [99]
*Aloe vera* (L.) Burm.f.	0.33	0.17	Leaf latex	Antibacterial, antifungal, and anti-oxidant activity [100] Antiviral activity [101] Anti-inflammatory activity [102] Skin hydration and anti-erythema effect [103] Skin permeation-enhancing effect [104] Wound healing activity [54] Melasma decreasing activity [96] Anti-psoriatic activity [105]
*Anacardium occidentale* L.	0.33	0.17	Pericarp	Anti-bacterial and anti-oxidant activity [106,107] Antifungal activity [108] Anti-inflammatory activity [109]
*Barringtonia acutangular* (L.) Garetn.	0.33	0.17	Stem bark	Anti-bacterial, antifungal, and anti-oxidant activity [110] Anti-inflammatory activity [111]
*Tinospora crispa* (L.) Miers ex Hook.f. & Thomson	0.33	0.17	Stem	Antibacterial, antifungal, and anti-oxidant activity [112] Anti-inflammatory activity [113] Wound healing activity [58]

## Data Availability

Not applicable.
